# Polyphenols improve non‐alcoholic fatty liver disease via gut microbiota: A comprehensive review

**DOI:** 10.1002/fsn3.4178

**Published:** 2024-06-07

**Authors:** Kimia Mohammadhasani, Mohammad Vahedi Fard, Ali Mottaghi Moghaddam Shahri, Zahra Khorasanchi

**Affiliations:** ^1^ Department of Nutrition, Food Sciences and Clinical Biochemistry, School of Medicine, Social Determinants of Health Research Center Gonabad University of Medical Sciences Gonabad Iran; ^2^ International UNESCO Center for Health‐Related Basic Sciences and Human Nutrition Mashhad University of Medical Sciences Mashhad Iran; ^3^ Department of Nutrition, School of Medicine Mashhad University of Medical Sciences Mashhad Iran

**Keywords:** gut microbiome, NAFLD, non‐alcoholic fatty liver disease, polyphenol

## Abstract

Polyphenols, natural micronutrients derived from plants, are valued for their anti‐inflammatory and antioxidant properties. The escalating global prevalence of non‐alcoholic fatty liver disease (NAFLD) underscores its status as a chronic progressive liver condition. Furthermore, the dysregulation of gut microbiota (GM) is implicated in the onset and progression of NAFLD through the actions of metabolites such as bile acids (BAs), lipopolysaccharide (LPS), choline, and short‐chain fatty acids (SCFAs). Additionally, GM may influence the integrity of the intestinal barrier. This review aims to evaluate the potential effects of polyphenols on GM and intestinal barrier function, and their subsequent impact on NAFLD. We searched through a wide range of databases, such as Web of Science, PubMed, EMBASE, and Scopus to gather information for our non‐systematic review of English literature. GM functions and composition can be regulated by polyphenols such as chlorogenic acid, curcumin, green tea catechins, naringenin, quercetin, resveratrol, and sulforaphane. Regulating GM composition improves NAFLD by alleviating inflammation, liver fat accumulation, and liver enzymes. Furthermore, it improves serum lipid profile and gut barrier integrity. All of these components affect NAFLD through the metabolites of GM, including SCFAs, choline, LPS, and BAs. Current evidence indicates that chlorogenic acid, resveratrol, quercetin, and curcumin can modulate GM, improving intestinal barrier integrity and positively impacting NAFLD. More studies are necessary to evaluate the safety and efficacy of naringenin, sulforaphane, and catechin.

## Graphical abstract

Gut microbiota functions and composition can be regulated by polyphenols such as chlorogenic acid, curcumin, green tea catechins, naringenin, quercetin, resveratrol, and sulforaphane. Regulating GM composition improves NAFLD by alleviating inflammation, liver fat accumulation, and liver enzymes. Furthermore, it improves serum lipid profile and gut barrier integrity.

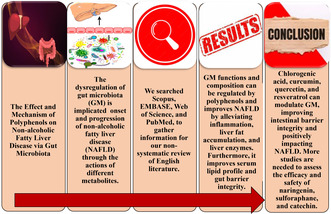

## 1 INTRODUCTION

Non‐alcoholic fatty liver disease (NAFLD) is a progressive liver disease related to metabolic disorders worldwide (Younossi et al., 2016). The prevalence of NAFLD is 46.9 patients per 1000 person‐years and is escalating at a highly concerning pace (Riazi et al., 2022). In 2015, the total number of deaths among the NAFLD population was evaluated at 1.27 million per year. It is predicted that by 2030, it will reach 1.83 million deaths per year (Estes et al., 2018). NAFLD prevalence is related to metabolic risk factors, such as obesity, diabetes, hypertension, and dyslipidemia (Chalasani et al., 2018; Samuel & Shulman, 2018). NAFLD patients are primarily asymptomatic before the diagnosis is made. However, some patients may complain of symptoms such as sleep disturbances, acanthosis nigricans, hepatomegaly, bloating, right upper quadrant discomfort, and complain of fatigue (Bacon et al., 1994; Khoonsari et al., 2017). This disease ranges from simple hepatic steatosis to non‐alcoholic steatohepatitis (NASH), NASH‐related cirrhosis, and hepatocellular carcinoma (Chalasani et al., 2018). So, recently NAFLD has been considered a significant health concern (Lazarus et al., 2022).

A balanced diet, including plant food supplemented with animal foods, can improve and prevent NAFLD (Plaz Torres et al., 2019). Dietary patterns like the Mediterranean diet that suggests a high intake of vegetables, fruits, legumes, and nuts, are rich in natural compounds with antioxidant and anti‐inflammatory properties and are effective in the prevention and management of NAFLD (Katsiki et al., 2022). However, adhering to the Western diet and diets with high amounts of saturated fats, red and processed meat, and sugar is a risk factor for NAFLD (Zhang, Powell, et al., 2021). Alleviating NAFLD by using natural compounds that are available, low‐cost, and safe has been considered an unmet need by physicians (Tarantino et al., 2021). Polyphenols are natural micronutrients derived from plants (Bravo, 1998). They are found in many food sources, including red fruits, vegetables, coffee, and green tea (D Archivio et al., 2007). Most polyphenols have antioxidant and anti‐inflammatory properties and play an essential role in the management of NAFLD (Rahimlou et al., 2024; Yu et al., 2016). Studies showed that polyphenols have been effective in the improvement of sarcopenia, a condition that contributes to the progression of NAFLD (Iwaki et al., 2023; Tarantino et al., 2023). The gut microbiota (GM) can control different diseases in the body (Y. Chen et al., 2021). Obesity, the main driver of NAFLD, is associated with gut and adipose tissue hormones (Lean & Malkova, 2016). GM can alter the secretion of these hormones and affect the progression of NAFLD (Koukias et al., 2017). However, losing weight and modifying gut hormonal levels through surgery are the main strategies for treating obesity‐related NAFLD (Finelli et al., 2014). In line with that, GM has the amazing ability to transform polyphenols into various compounds that possess the potential to impact the host (Selma et al., 2009). These compounds are essential in the prevention and treatment of pathological diseases, as well as certain non‐communicable chronic diseases (Afshari et al., 2019; Aron‐Wisnewsky et al., 2020). Animal studies indicate that various kinds of polyphenols have the potential to reverse or enhance characteristics associated with NAFLD by influencing the GM and modulating the gut–liver axis (Ji et al., 2023).

The complicated connection between the gut and liver is established via the portal vein and the mesenteric lymphatic system, called the gut–liver axis (Albillos et al., 2020). It highlights the significant relationship between the GM and the progression of NAFLD. In general, GM consists of six phyla including *Bacteroidetes*, *Firmicutes*, *Fusobacteria*, *Actinobacteria*, *Proteobacteria*, and *Verrucomicrobia*, while *Bacteroidetes* and *Firmicutes* are predominant (Hou et al., 2022). Dysbiosis is characterized by a decrease in microbial diversity, the depletion of beneficial bacteria like *Bacteroides* strains, and an increase in bacteria such as *Proteobacteria* (Humphreys, 2020). An imbalance in GM can lead to impaired liver function, which ultimately contributes to the progression of NAFLD (Ma & Wu, 2017). In the present study, we aimed to review the beneficial effect of specific polyphenols, such as chlorogenic acid (CGA), curcumin, green tea catechins, naringenin, quercetin, resveratrol, and sulforaphane, on NAFLD by influencing the composition of GM.

## 2 MECHANISM BETWEEN GM AND NAFLD

**FIGURE 1:**
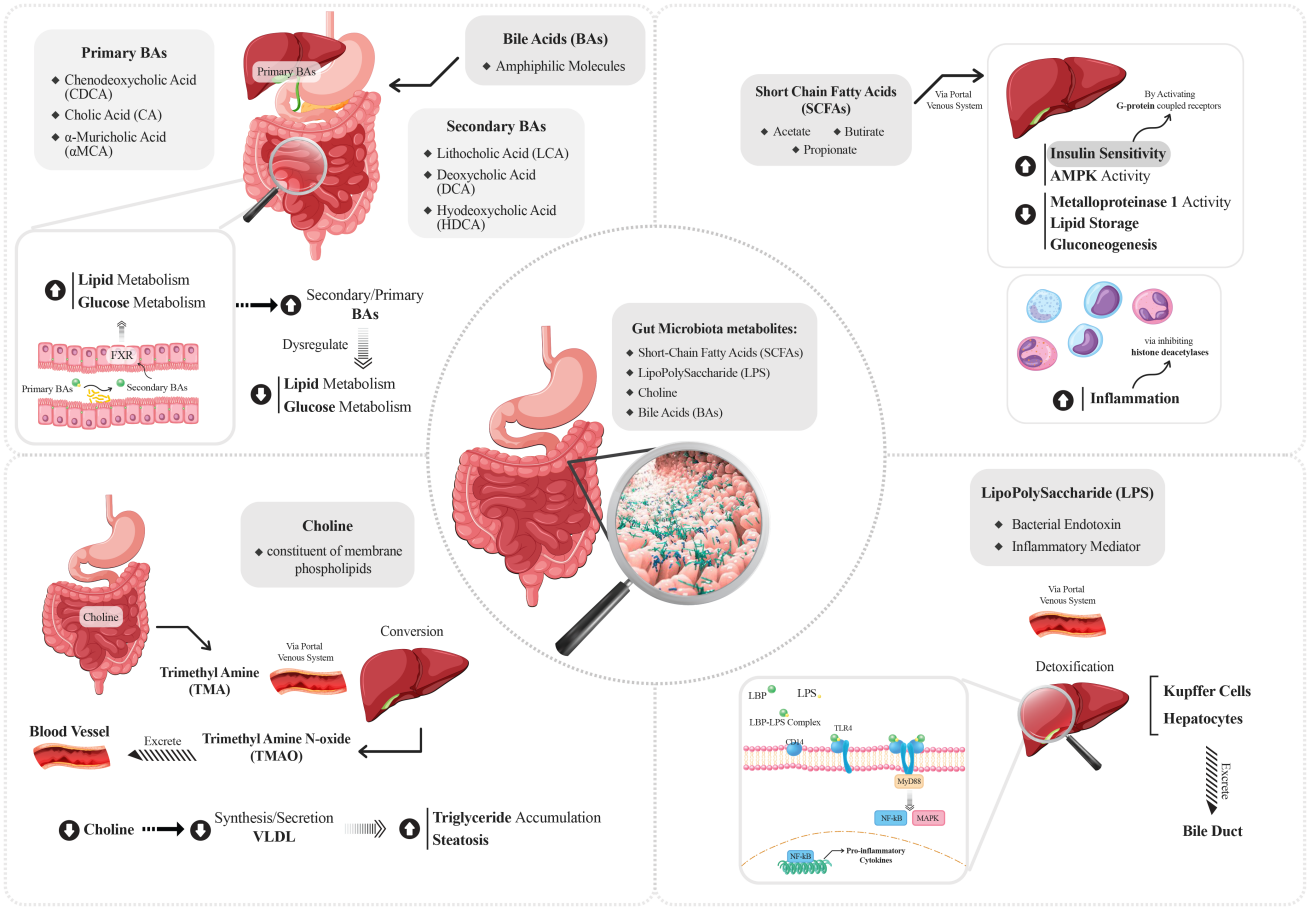
A comprehensive framework model illustrating the gut–liver axis and related mechanisms.

GM plays a pivotal role in NAFLD through its metabolites, such as SCFAs, LPS, choline, and BAs (Figure 1). In other words, they attracted significant attention for affecting lipid metabolism, inflammation, apoptosis, and fibrosis during NAFLD development.

### 2.1 Short‐chain fatty acids

Most nutrients undergo digestion and absorption within the intestinal tract. Gut microorganisms play a vital role in this process by producing SCFAs via fermentation of dietary fibers (Silva et al., 2020). Gut‐derived SCFAs are transported to the liver through the hepatic portal vein and could be known as a signaling connection between gut dysbiosis and the NAFLD progression. Affecting insulin sensitivity and nutrient absorption by activating G‐protein‐coupled receptors were noticed in different studies (Aragonès et al., 2019; Koh et al., 2016). Also, SCFAs regulate immune functions and alleviate liver inflammation via inhibiting histone deacetylases (Schilderink et al., 2013). In addition to regulating the production of inflammatory cytokines like a terminal product of the toll‐like receptor 4 (TLR4) signal pathway and tumor necrosis factor alpha (TNF‐α), SCFAs play a vital role in liver inflammation and fibrosis. They enhance the mRNA expression of tissue inhibitors of metalloproteinase 1 in activated hepatic stellate cells (HSCs) and suppress the apoptosis of HSCs, further contributing to these conditions (Li, Deng, et al., 2021; Stojsavljević et al., 2014). Furthermore, SCFAs could activate activated protein kinase (AMPK) which downregulates acetyl‐coenzyme A carboxylase and sterol receptor element‐binding protein 1c, whereas it upregulates peroxisome proliferator‐activated receptors α (PPARα). This process inhibits lipogenesis and promotes the oxidation of fatty acids in the liver (Liu et al., 2020).

### 2.2 Lipopolysaccharide

LPS is a bacterial endotoxin and a potent inflammatory mediator. It affects liver fat deposition, liver damage, and NAFLD (Sharifnia et al., 2015). The portal venous system transfers LPS into the liver and detoxifies in it. Furthermore, elevated intestinal permeability and small intestinal bacteria overgrowth cause bacterial translocation and LPS. In the liver, Kupffer cells and hepatocytes take up LPS and excrete it to the bile duct eventually (Vespasiani‐Gentilucci et al., 2015). NAFLD patients have high LPS portal/peripheral levels (Carpino et al., 2020). LPS binds to the LPS‐binding protein (LBP) and creates the LBP–LPS complex. Subsequently, the complex is transferred to either a membrane‐bound or soluble cluster of differentiation 14 (CD14). CD14 selectively binds to TLR4, leading to the activation of the adaptor molecule myeloid differentiation factor 88 (MyD88). MyD88 then activates downstream pathways, including mitogen‐activated protein kinase (MAPK) and nuclear factor‐κB (NF‐κB). Notably, TLR4 signaling is recognized as a pivotal mechanism. In normal liver, hepatic cells demonstrate minimal expression of toll‐like receptors (TLRs), which shows high tolerance to TLR ligands (An et al., 2022). Activation of the LPS‐TLR4 signaling pathway causes proinflammatory molecules release, like TNF‐α and IL‐6, which assume an essential role in the pathogenesis of NAFLD (Kawaratani et al., 2013; Roh & Seki, 2013). Liver cells, such as hepatocytes, cholangiocytes, HSCs, and Kupffer cells, express TLR4. The expression of TLR4 progresses to the stage of fibrosis (Vespasiani‐Gentilucci et al., 2015).

### 2.3 Choline

Choline is a nutrient with considerable attention in the progression of NAFLD. Choline, an important constituent of membrane phospholipids, assumes an essential role in cholesterol and lipid metabolism within the liver. By facilitating the transportation of fats in the form of phospholipids, choline contributes to various physiological processes and helps prevent the pathological accumulation of fat in hepatic tissue (Corbin & Zeisel, 2012). GM metabolizes choline and produces trimethylamine (TMA). TMA is transformed into trimethylamine‐N‐oxide (TMAO) after absorption by the host in the liver. TMAO causes inflammation, liver damage, and NAFLD progression (Fennema et al., 2016; Spencer et al., 2011). Elevated levels of blood TMAO considered a predictive indicator for increased TMA production, thereby indirectly reflecting alterations in choline and phosphatidylcholine metabolism. Insufficient choline levels can impair the synthesis and secretion of very low‐density lipoproteins that leads to triglyceride (TG) accumulation and steatosis in the liver (Mehedint & Zeisel, 2013).

### 2.4 Bile acids

Bile acids (BAs) are amphiphilic molecules first synthesized from cholesterol within hepatocytes and then metabolized by GM (Jones et al., 2008). Primary BAs like cholic acid (CA), α‐muricholic acid (αMCA), and chenodeoxycholic acid (CDCA) are finally converted into secondary BAs, such as lithocholic acid (LCA), deoxycholic acid (DCA), and hyodeoxycholic acid (HDCA) (Zeng et al., 2019). BAs effect on NAFLD by cholesterol and glucolipid metabolism pathways (Chow et al., 2017). They induce NAFLD by farnesoid X receptor (FXR) as a signaling factor (Shao et al., 2021). FXR, a nuclear hormone receptor, assumes a vital role in the regulation of both lipid and glucose metabolism (McMahan et al., 2013). Increased ratio of secondary to primary BAs dysregulate glucose and lipid metabolism through FXR (Abdelmalek, 2021; Hrncir et al., 2021). Therefore, NAFLD may be influenced by GM through changing intestinal BAs and FXR signaling pathways.

### 2.5 Intestinal barrier

The intestinal barrier plays a pivotal role in preventing micro‐organism invasion into the lumen. Elevated intestinal permeability through alteration of intestinal barrier function is related to the development of liver damage (De Munck et al., 2020; Farré & Vicario, 2016). Studies suggest that 39.1% of NAFLD cases had enhanced intestinal permeability (Farré & Vicario, 2016). The intestinal barrier integrity is preserved by the epithelial layer (Bermudez‐Brito et al., 2012). The transmembrane proteins forming tight junctions (TJs) include the TJ‐associated proteins, specifically junctional adhesion molecules and Claudins (Suzuki, 2020). The first TJ‐associated protein is zonula occludens (ZO‐1), which is known as an essential marker for the detection of intact cell‐to‐cell contacts and the evaluation of TJ integrity (Stevenson et al., 1986). Other major transmembrane proteins are Claudin and Occludin, which regulate paracellular diffusion (Chelakkot et al., 2018). TJ protein expression is modulated by the immune system. The microbiome composition molds the immune system (Isaacs‐Ten et al., 2020; Portincasa et al., 2021).

## 3 METHOD

This narrative review aimed to investigate the effect of polyphenols on NAFLD via GM. A search of English language literature was conducted using Web of Science, Scopus, PubMed, and EMBASE. We searched up to December 19th, and no restriction in time was applied. Articles were searched using the keywords “Non‐alcoholic Fatty Liver Disease”, “NAFLD,” “Nonalcoholic Steatohepatitis,” “Chlorogenic Acid,” “Curcumin,” “Green Tea Catechins,” “Naringenin,” “Quercetin,” “Resveratrol,” “Sulforaphane,” “Polyphenol,” “Gastrointestinal Microbiome,” “Gut Microbiome,” and “Intestinal Microbiota.” We integrated articles that are relevant to the subject matter into our study. Furthermore, we included extra papers that the authors were familiar with.

## 4 RESULTS

### 4.1 Chlorogenic acid

CGA is a significant component of coffee (Tajik et al., 2017). Studies showed the advantageous effects of CGA on obesity, brain function, and irritable bowel syndrome via modulating GM (Wang et al., 2019; Zheng et al., 2023). Gut–liver axis is affected by different polyphenols (Wang et al., 2021). So, CGA effects on GM and NAFLD should be assessed (Table 1).

**TABLE 1:** A review of studies that examined the effect of polyphenols on NAFLD via gut microbiota.

Authors	Dose & Duration	Population	Effect	Changes in gut microbiota
Chlorogenic acid				
Li et al. (2023)	90 mg/kg geniposide +1.34 mg/kg chlorogenic acid p.o. 4 weeks	C57BL/6 mice	↓ liver TG ↓ serum TC, TG, ALT, and AST ↓ HOMA‐IR ↓ serum fast blood glucose and fasting insulin	↓ *Deferribacteres*, *Proteobacteria*, and *Firmicutes* ↓ *Desulfovibrio*, *Roseburia*, *norank_f_Lachnospiraceae*, and *unclassified_f_Lachnospiraceae* ↑ *Tenericutes*, *Verrucomicrobia*, and *Bacteroidetes* ↑ *Akkermansia*, *Ruminiclostridium_9*, and *Bacteroides*
Shi et al. (2021)	60 mg/kg p.o. 12 weeks	C57BL/6 mice	↑ expression of ZO‐1 and Occludin ↓ expression of IL‐6 and TNF‐α in the liver ↓ activation of the TLR4 signaling pathway in the liver ↓ LPS levels in portal vein ↑ GLP‐1 levels in portal vein	↑ *Bifidobacterium* ↓ *Escherichia coli*
Leng et al. (2022)	3.3 mg/kg chlorogenic acid +266.67 mg/kg *Atractylodes macrocephala* polysaccharide + and 45 mg/kg geniposide intragastric 4 weeks	C57BL/6 mice	↓ hepatic lipopolysaccharide‐binding protein Rarer positive staining of F4/80 ↓ mRNA expression of MyD88 and CD14 ↓ hepatic content of IL‐1β and TNF‐α ↑ ZO‐1 and Occludin in the colon tissue	↑ *Erysipelatoclostridium* and *Jeotgalicoccus*
Zhang, Powell, et al. (2021)	200 and 400 mg/kg p.o. 12 weeks	Kunming mice	↓ serum levels of trimethylamine and trimethylamine N‐oxide ↑ colonic SCFAs	↓ *Firmicutes* ↑ *Bacteroidetes* ↑ *Bacteroides*
Mansour et al. (2020)	200 Mg Chlorogenic Acid +200 Mg Caffeine 12 Weeks	Patients With Non‐Alcoholic Fatty Liver	Weight reduction	Not significant
Curcumin				
Feng et al. (2017)	200 mg/kg p.o. 4 weeks	Sprague Dawley male rats	↓ liver weight, and hepatic lipid contents ↓ serum ALT and AST levels ↓ plasma lipopolysaccharide and diamine oxidase circulating levels ↓ TNFα levels ↓ Tight‐junction‐width of the gut barrier and	↑ *Lactobacillus* ↓ *Bacteroides*, *Mucispirillum*, *Blautia*, *Coprococcus*, *Anaerotruncus*, *Ruminococcus*, *Allobaculum*, and *Helicobacter*
Hong et al. (2022)	0.1% w/w in the normal diet 24 weeks	CD‐1 male mice	↓ TC, TG, LDL‐C, and HDL‐C ↓ ALT and AST ↑ ZO‐1 and Occludin ↓ serum LPS, DAO, D‐lactate levels ↓ serum levels of IL‐18, IL‐1β, IL‐6, and TNF‐α	↑ *Verrucomicrobia* and *Akkermansia* ↓ *Proteobacteria* ↓ *Firmicutes* to *Bacteroidetes* ratio
Li, Deng, et al. (2021)	200 mg/kg/day 14 weeks	Male Sprague Dawley rats	↓ serum levels of ALT, AST, TG, LDL‐C, and glucose ↑ HDL‐C ↓ serum levels of IL‐23, TNF‐a, IL‐6, IL‐1β, OX40, and interferon‐inducible protein‐10. ↓ lipid vacuoles and lipid accumulation in liver Improved intestinal barrier integrity	↓ *Lactobacillus*, *Corynebacterium*, *Akkermansia*, and *Helicobacter* ↓ some bacteria from *Ruminococcaceae*, *Lachnospiraceae*, *Corynebacterium*, and *Clostridiales families*
Green tea catechins				
Ning et al. (2020)	50 mg/kg 2 weeks	C57BL/6J mice	↓ TG and TC ↓ the ALT and HE scores ↓iron loading ↓ the neutral lipid area ↓ the fibrosis scores	↓ *norank_f__Ruminococcaceae*, *Ruminiclostridium_9*, *Butyricimonas*, *Odoribacter*, *Parasutterella*, *Bifidobacterium* ↑*Unclassified_f__Ruminococcacea*, *Alloprevotella*, *Bacteroides*
Dey et al. (2020)	0.3% of diet 8 weeks	C57BL/6J mice	Improves obesity, insulin resistance, NASH, liver injury, endotoxin‐TLR4‐NFκB inflammation, gut barrier dysfunction, and intestinal inflammation	↑ *Bacteroidetes* and *Verrucomicrobia* ↓ *Firmicutes* and *Tenericutes* Order level: ↓ *Clostridiales* and *Mollicutes RF39* ↑ *Anaeroplasmatales* ↓ *Ruminiclostridium*, *Clostridium cluster 1*, *Blautia*, *Lachnospiraceae NK4A136*, *Acetatifactor*, *Lachnospiraceae UCG‐006*, and *Lachnoclostridium* ↑ *Akkermansia*
Ushiroda et al. (2019)	0.32% of diet 8 weeks	C57BL/6N mice	↑ Serum levels of predominant primary CA and CDCA ↑ α‐MCA and β‐MCA ↓ Tauro‐CA, Tauro‐α‐MCA, Tauro‐β‐MCA, and Tauro‐DCA ↑ serum CA/(CA + Tauro‐CA) ↓ serum DCA/(CA + DCA)	↑ Allobaculum, Adlercreutzia, Akkermansia, Parabacteroides, f_Erysipelotrichaceae, and g‐Clostridium ↓ Mucisprillium, Ruminococcus, f_Lachnospiraceae, f_Desulfovibrionaceae, and Anaerotruncus
Cremonini et al. (2018)	EC (2–20) mg/kg body weight 2 weeks	C57BL/6J mice	Not significant	Not significant
Naringenin				
Mu et al. (2020)	0.07% NAR 8 weeks	C57BL/6J mice	↓ TC, HDL‐C, and LDL‐C	↑ *uncultured_bacterium_f_Muribaculaceae*, *Parasutterella*, *Lachnospiraceae_NK4A136_group*, *Butyricicoccus*, *Alloprevotella*, and *Allobaculum* ↓ *Fusobacterium*, *Coriobacteriaceae_UCG‐002*, *Faecalibaculum*, and *Campylobacter*
Yu et al. (2023)	0.1% naringenin of diet 16 weeks	SPF C57BL/6J mice	↓ body weight, liver weight, epididymal fat weight, subcutaneous fat weight of groin, and brown fat weight ↓ TG and TC in liver and blood ↓ gene expression of IL‐1β, IL‐6, TNF‐α, and F4/80 ↓ Myeloperoxidase activity ↑ expression of Occludin and Claudin‐2	↓ *Proteobacteria*, *Epsilonproteobacteria* ↓ *Rikenellaceae*, *Helicobacteraceae*, and *Peptostreptococcaceae* ↓ *Romboutsia*, *Alistipes*, *Faecalibaculum*, *Lachnospiraceae_NK4A136_group*, and *Desulfovibrio* ↓ *Parasutterella* and *Bacteroidales_S24‐7_group*
Quercetin				
Shi et al. (2022)	0.5% isoquercetin 0.5% quercetin 15 weeks	C57BL/6 mice	↑ indole‐3‐propionic acid, indole acetic acid	↑ *Lactobacillus*, *Bifidobacterium*, and *Akkermansia* ↓ *Streptococcus*, *Anaerostipes*, and *Lachnoclostridium*
Porras et al. (2017)	0.05% of diet aglycone quercetin 16 weeks	C57BL/6J mice	↓ activation of (TLR‐4)‐NF‐κB pathway, expression of pro‐inflammatory genes, reticulum stress pathway improved gut barrier dysfunction	↓ *Proteobacteria* and *Firmicutes/Bacteroidetes* ratio ↓ *Deltaproteobacteria* ↑ Betaproteobacteria, Erysipelotrichi, and Bacteroidia ↓ *Helicobacter* and *Desulfovibrio* ↑ *Sutturella*, *Allobaculum*, and *Flavobacterium*
Juárez‐Fernández et al. (2021)	Aglycone quercetin 37.5 mg/kg p.o., *Akkermansia muciniphila* 2 × 108 colony‐forming units p.o. + aglycone quercetin 37.5 mg/kg p.o. 3 weeks	Wistar rats	↓ plasma concentrations of deoxycholic acid and secondary hyodeoxycholic acid ↑ cholic acid /deoxycholic acid ratio ↑ primary bile acids and primary α‐muricholic acid plasma concentrations ↓ hepatic expression of PPAR‐α, IL‐6, and of IL‐1β	↑ *Cyanobacteria* ↓ *Actinobacteria*, *Coprobacillus* and *Roseburia*
Resveratrol				
Du et al. (2021)	50 mg/kg p.o. 4 weeks	C57BL/6J mice	↓ fasting blood glucose, and HOMA‐IR ↓ liver TG, fat weight, body weight, and oil red O staining ↓ mTOR, phospho‐mTOR, and peroxisome proliferator activated receptor gamma ↑ phospho‐insulin receptor substrate 1	↓ *Firmicutes*, *Ruminococcaceae*, *Lachnospiraceae*, *Hydrogenoanaerobacterium*, *Anaerotruncus*, *Oscillibacter*, *Peptococcus*, *Clostridium XlVb*, *Flavonifractor*, *Intestinimonas*, and *Pseudoflavonifractor* ↑ *Bacteroidetes*, *Porphyromonadaceae*, *Allobaculum*, *Parasutterella*, and *Barnesiella*
Zhao et al. (2022)	50 mg/kg mg/kg p.o. polydatin and resveratrol 10 weeks	C57BL/6J mice	↑ fecal levels of valeric acid and caproic acid	↑ *Bifidobacterium* and *Butyricimonas* ↑ *Muribaculum*, *Desulfovibrio*, and *Rinkenella* ↓ *Lactobacillus*
Chen et al. (2020)	50, 100 mg/kg p.o. 6 weeks	Sprague Dawley rats	↓ plasma lipopolysaccharide ↑ expression of Occluding and ZO‐1 ↓ expression of cannabinoid receptor 1 ↑ expressions of cannabinoid receptor 2	↓ Desulfovibrio ↑ Akkermansia muciniphila, Ruminococcaceae, and Lachnospiraceae
Wang et al. (2020)	300 mg/kg p.o. 16 weeks	C57BL/6 J mice	↓ liver oxidative stress and inflammation ↓ body weight and fat accumulation Improving gut barrier permeability	↑ *Bacteroides*, *Allobaculum*, and *Blautia* ↓ *Alistipes*, *Lachnospiraceae_NK4A316_group*, and *Desulfovibrio*
Ke et al. (2023)	20, 100 mg/kg p.o. (hydroxypropyl‐beta‐cyclodextrin embedded 20, 100 mg/kg resveratrol p.o.) 13 weeks, 5 weeks	C57BL/6 mice	↑ activation of the AMPK pathway	↓ *Firmicutes/Bacteroidetes* ration and *Verrucomicrobia* ↑ *Bacteroidetes* ↑ *Prevotella* ↓ *Facklamia*
Gao et al. (2023)	50 mg/kg p.o. 8 weeks	C57BL/6J mice	↓ TG, IL‐6, lipopolysaccharide, and mRNA levels of NF‐κB and TLR4	↓ Alistipes, Desulfovibrio, and Lachnospiraceae_NK4A136_group
Milton‐Laskibar et al. (2021)	30 mg/kg p.o. 8 weeks	Wistar rats	Not significant	↑ *Blautia* and *Lactococcus*
Sulforaphane				
Xu et al. (2023)	15, 30 mg/kg p.o. 12 weeks	C57BL/6 mice	↑ expression of ZO‐1 and Claudin‐4 genes, ↑ IL‐10/IL‐6 ratio in the liver ↓ serum lipopolysaccharide, ↓ TNF‐α, IL‐1β, and IL‐6 genes expression ↓ C‐C motif ligand 2 and C‐C motif ligand 4 ↓ expression of MyD88, NF‐κB, TLR4, and endoplasmic reticulum stress, c‐Jun N‐terminal kinase, tumor necrosis factor‐receptor‐associated factor 2, and C/EBP‐homologous protein ↓ TLR4/NF‐κB colonic activation	↑ *Butyricicoccaceae*, *Butyricicoccus*, *Allprevotella*, *Akkermansia*, *Bifidobacterium*, and *Lachnospiraceae*_*NK4A136*_*group* ↓ *Desulfovibrionaceae*, *Streptococcaceae* ↓ *Lactobacillus*, *Desulfovibrio*, *Blautia*, *Lactococcus* ↓ *Firmicutes*/*Bacteroidota* ratio
Xu et al. (2021)	25 mg/kg p.o.6 weeks	C57BL/6 mice	↑ serum Indole‐3‐acetic acid	↑ *Bacteroides* and *Bifidobacterium* ↑ *Firmicutes/Bacteroidetes* ↓ *Deferribacteres*

Analyzing 50 male C57BL/6 mice, Li et al. reported that CGA plus geniposide supplementation improves NASH by regulating GM. Liver TG levels and TG, serum total cholesterol (TC), alanine transaminase (ALT), and aspartate aminotransferase (AST) levels were decreased after supplementation. Furthermore, homeostatic model assessment for insulin resistance (HOMA‐IR) as well as fasting insulin levels and serum fast blood glucose were reduced, too. In terms of GM, the intestinal abundance of *Deferribacteres*, *Firmicutes*, and *Proteobacteria* was attenuated, while the abundance of *Tenericutes*, *Bacteroidetes*, and *Verrucomicrobia* was increased at the phylum level. At the genus level, the abundance of *Desulfovibrio*, *Roseburia*, *norank_f_Lachnospiraceae*, and *unclassified_f_Lachnospiraceae* was downregulated, whereas the abundance of *Akkermansia*, *Ruminiclostridium_9*, and *Bacteroides* was upregulated (Li et al., 2023).

Shi et al. used C57BL/6 mice to assess the effect of CGA on NAFLD. They revealed that CGA supplementation increases the expression of Occludin and ZO‐1. Also, it reduced the expression of TNF‐α, IL‐6, and the activation of the TLR4 signaling pathway in the liver. LPS levels were decreased in the portal vein, whereas glucagon‐like peptide 1 (GLP‐1) levels were enhanced in it. According to GM composition, CGA attenuated the abundance of *Escherichia coli* and elevated the abundance of *Bifidobacterium* (Shi et al., 2021).

Leng et al. evaluated male mice and indicated that consuming CGA with *Atractylodes macrocephala* polysaccharide and geniposide improves NASH. Rarer positive staining of F4/80 (a unique marker of murine macrophages) in the liver and lower hepatic lipopolysaccharide‐binding protein was observed after supplementation. Also, mRNA expression of MyD88 and CD14 and the hepatic content of TNF‐α and IL‐1β were reduced. Intestinal gut barrier integrity was improved by restoring ZO‐1 and Occludin proteins in the colon tissue. According to GM composition, it elevated the relative abundance of *Jeotgalicoccus* and *Erysipelatoclostridium* (Leng et al., 2022).

Zhang et al. analyzed the effect of CGA intake on liver damage caused by high L‐carnitine intake. They found that CGA modifies GM composition and increases the abundance of *Bacteroidetes* and *Bacteroides*, whereas it attenuates the abundance of *Firmicutes*. L‐carnitine increased serum TMA and TMAO levels. *Firmicutes* were positively associated with serum TMAO and TMA levels, whereas *Bacteroidetes* were negatively associated. *Bacteroides* were negatively associated with serum TMAO and TMA levels at the genus level. Also, L‐carnitine reduced colonic SCFAs, and CGA supplementation reversed it. *Firmicutes* were negatively associated with propionic acid, acetic acid, butyric acid, isobutyric acid, isovaleric acid, and valeric acid. In contrast, *Bacteroidetes* and *Bacteroides* were positively associated with isovaleric acid, valeric acid, and isobutyric acid (Zhang, Shi, et al., 2021).

In a human study analyzing 26 NAFLD patients, the effect of 200 mg CGA plus 200 mg caffeine supplementation on NAFLD was assessed. It caused weight reduction among these patients and raised the number of *Bifidobacterium* but it did not increase significantly (Mansour et al., 2020). This study used a minimum dosage of supplementation for patients, which may lead to insignificant results. Also, the beneficial effects of caffeine are still controversial and need more studies (Calabrò et al., 2020).

Based on the studies, we found that consuming CGA supplements can modulate the GM and intestinal barrier integrity, resulting in a positive effect on NAFLD. It can potentially improve inflammatory factors, lipid profile, liver enzymes, bile acids, and insulin resistance (IR).

### 4.2 Curcumin

Curcumin is a polyphenol primarily extracted from Curcuma longa (Aggarwal et al., 2003). It has antioxidant, antidiabetic, anti‐inflammatory, hepatoprotective, and anticancer effects, which are considered therapeutic targets (Slika & Patra, 2020). Research has shown that curcumin can affect the diversity of GM and modulate it (Scazzocchio et al., 2020). Therefore, it may have positive effects on NAFLD (Table 1).

Feng et al. in their animal study assessed the effect of curcumin on GM changes in high‐fat diet (HFD) induced NAFLD among male rats. Hepatic ectopic fat deposition, liver weight, serum TG, and serum liver enzyme levels (ALT and AST) were reduced after curcumin supplementation among these animals. Furthermore, it enhanced intestinal barrier integrity by elevating Occludin and ZO‐1 expression and also circulating levels of diamine oxidase. Plasma LPS and TNF‐α levels were reduced by curcumin supplementation. After curcumin intake, the microbiota composition underwent alterations. *Lactobacillus* was enriched whereas the relative abundance of *Bacteroides*, *Mucispirillum*, *Blautia*, *Anaerotruncus*, *Coprococcus*, *Ruminococcus*, *Allobaculum*, and *Helicobacter* were reduced (W. Feng et al., 2017).

In the study reported by Hong et al., bisphenol A (BPA)‐induced hepatic steatosis in male mice was assessed. Curcumin intake prevented disordered arrangement of liver plates steatosis and steatosis of liver cells. Curcumin reduced serum liver enzymes and also improved serum lipid profile, liver inflammation, and gut barrier integrity significantly. In terms of GM, BPA induced significant alterations in its composition, while curcumin supplementation ameliorated them. The abundance of *Verrucomicrobia* was significantly lower in the BPA group than in the curcumin‐treated group and control group. The predominant genus comprising *Verrucomicrobia*, *Akkermansia*, was negatively related to serum ALT, AST activity, and IL‐18, TNF‐α, IL‐1β, IL‐6, LPS, low‐density lipoprotein cholesterol (LDL‐C) levels, TC, and liver TC content. Moreover, it was positively related to serum high‐density lipoprotein cholesterol (HDL‐C) levels. Additionally, the BPA group demonstrated elevated abundance of *Proteobacteria* and a higher *Firmicutes* to *Bacteroidetes* ratio in comparison to the control group. Curcumin intake attenuated them significantly. *Bacteroidetes* enrichment was positively related to serum ALT, AST activity, and IL‐18, IL‐1β, TNF‐α, LPS, TG, and LDL‐C levels. It was negatively associated with serum HDL‐C levels. Also, the richness of *Firmicutes* serum was positively related to ALT, AST activity, and TG levels as well as liver TC content (Hong et al., 2022).

Using HFD‐induced NAFLD rats, Li et al. found a significant reduction in hepatic fat deposition, inflammatory cytokines, liver enzyme levels, and serum glucose levels after curcumin supplementation. Also, it improved serum lipid profile and intestinal barrier integrity. Curcumin mediated GM and ameliorated inflammation and hepatic steatosis. They found that *Lactobacillus*, *Corynebacterium*, *Akkermansia*, *Helicobacter*, and also some other bacteria from *Ruminococcaceae*, *Lachnospiraceae*, *Corynebacterium*, and *Clostridiales* in curcumin group were positively related to serum levels of IL‐1β, TNF‐α, IL‐6, TC, LDL‐C, and TG, and negatively associated with HDL‐C. Curcumin reduced these bacteria. However, *Stomatobaculum*, *Ruminococcus*, and some bacteria from *Firmicutes* phylum, *Ruminococcaceae*, *Porphyromonadaceae*, and *Lachnospiraceae* families were negatively associated with glucose, IL‐23, IL‐1β, TNF‐α, IL‐6, LDL‐C, TC, TG, AST, ALT, OX40, and interferon‐inducible protein‐10 (R. Li, Yao, et al., 2021). These studies showed that curcumin supplementation modulates GM and intestinal barrier integrity. It can improve inflammatory factors, lipid profile, liver enzymes, and glucose levels. Therefore, curcumin has a positive effect on NAFLD.

### 4.3 Green tea catechins

Green tea catechins are natural antioxidants with many health‐promoting effects (Y. Suzuki et al., 2012). The main catechins of green tea are epicatechin, epigallocatechin, epicatechin gallate, and epigallocatechin gallate (EGCG) (Musial et al., 2020). They can impact the composition of the GM by either enhancing the growth of beneficial species or inhibiting the growth of harmful ones (Pérez‐Burillo et al., 2021). Therefore, evaluating their effect on the gut–liver axis would be necessary (Table 1).

Ning et al. studied C57BL/6J mice with NASH. They concluded that gavage or injection of 50 mg/kg EGCG for two weeks may have beneficial effects on NASH through balancing the gut microbiome and specific enzymes from genera. EGCG supplementation reduced *norank_f__Ruminococcaceae*, *Ruminiclostridium_9*, *Butyricimonas*, *Odoribacter*, *Parasutterella*, and *Bifidobacterium*. However, it evaluated *Unclassified_f__Ruminococcaceae*, *Alloprevotella*, and *Bacteroides*. Analyzing bacteria showed that microbial genera, such as *norank_f__Ruminococcaceae*, were positively associated with TG and TC. Furthermore, *Ruminiclostridium_9* had a positive relationship with hematoxylin–eosin scores (evaluates the histological morphology and lipid deposition within liver tissue), and *Parasutterella* had the same relationship with the ALT. Moreover, iron loading had a significant negative association with *Unclassified_f__Ruminococcaceae*. *Alloprevotella* and *Bacteroides* were negatively associated with the neutral lipid area. However, *Ruminiclostridium _9*, *Butyricimonas*, and *Odoribacter* had a significant positive association with the neutral lipid area. Also, the fibrosis score had a negative relationship with *Alloprevotella* and a positive relationship with *Bifidobacterium* (Ning et al., 2020).

Dey et al. studied C57BL/6J mice with NASH induced by HFD for 8 weeks. They found that consuming EGCG improves obesity, IR, NASH, liver injury, endotoxin‐TLR4‐NFκB inflammation, gut barrier dysfunction, and intestinal inflammation. In terms of GM, it increased the abundance of *Bacteroidetes* and *Verrucomicrobia* while decreasing the abundance of *Tenericutes* and *Firmicutes* at the phylum level. Furthermore, EGCG intake elevated the abundance of *Anaeroplasmatales* and decreased the abundance of *Clostridiales* and *Mollicutes RF39*. At the genus level, it reduced the abundance of *Ruminiclostridium*, *Clostridium cluster 1*, *Blautia*, *Lachnospiraceae NK4A136*, *Acetatifactor*, *Lachnoclostridium*, and *Lachnospiraceae UCG‐006* whereas increased the abundance of *Akkermansia* (Dey et al., 2020).

Ushiroda et al. in their study on HFD‐fed C57BL/6N mice, assessed the effect of EGCG supplementation on serum bile acids dysregulation and NAFLD. They found that EGCG intake may improve serum bile acid dysregulation via regulating GM. EGCG significantly increased the abundance of *Allobaculum*, *Adlercreutzia*, *Akkermansia*, *Parabacteroides*, *f_Erysipelotrichaceae*, and *g‐Clostridium* genera and reduced the abundance of *Mucisprillium*, *Ruminococcus*, *f_Lachnospiraceae*, *f_Desulfovibrionaceae* and *Anaerotruncus*. There was a significant correlation between higher abundance of *Allobaculum*, *Akkermansia*, and *Adlercreutzia* and higher serum levels of predominant primary CA and CDCA. Also, a significant positive relationship was observed between α‐MCA and β‐MCA and the abundance of *g‐Clostridium*, and *f_Erysipelotrichaceae*. However, the abundance of *Lachnospiraceae*, *Anaerotruncus*, and *Desulfovibrionaceae* was negatively related to the composition of CDCA and CA, while were positively associated with Tauro‐CA, Tauro‐α‐MCA, Tauro‐β‐MCA, and Tauro‐DCA. Furthermore, a significant positive association was found between Parabacteroides, *Allobaculum*, *Adlercreutzia*, and *Akkermansia* and serum CA/(Tauro‐CA + CA). Another significant positive association was observed between *Ruminococcus*, *f_Lachnospiraceae*, *Anaerotruncus*, and *f_Desulfovibrionaceae* and serum DCA/(CA + DCA) (Ushiroda et al., 2019).

In another study, Cremonini et al. analyzed C57BL/6J mice. They did not find any significant relationship between y (−)‐epicatechin supplementation for two weeks and normalization of HFD‐induced dysbiosis in steatosis (Cremonini et al., 2018).

In certain studies, supplementation of green tea catechins has shown a positive effect on inflammatory factors, lipid profile, liver enzymes, IR, and liver fat deposition by modulating the GM. However, more studies are necessary to confirm these results.

### 4.4 Naringenin

Naringenin is a flavonoid in citrus fruits, cocoa, and tomatoes (Salehi et al., 2019; Sánchez‐Rabaneda et al., 2003). It has antioxidant, anticancer, antidiabetic, anti‐inflammatory, and cardioprotective activities (Alam et al., 2014; Choi et al., 2020). Furthermore, naringenin has been found to improve GM composition and gut barrier function (Duda‐Chodak, 2012). In terms of NAFLD, the effects of naringenin on the gut–liver axis should be considered (Table 1).

In gut bacteria analysis, Mu et al. showed that naringenin supplementation modulated the gut bacteria compositional structure. It can also facilitate lipogenesis, liver lipid accumulation, and reduced plasma biochemical parameters in mice with NAFLD by attenuating harmful and elevating beneficial bacteria. In terms of GM, some bacteria from *uncultured_bacterium_f_Muribaculaceae*, *Parasutterella*, *Lachnospiraceae_NK4A136_group*, *Butyricicoccus*, *Alloprevotella*, and *Allobaculum* genera had a negative relationship with Serum LDL‐C, HDL‐C, TC levels. *Fusobacterium*, *Coriobacteriaceae_UCG‐002*, *Faecalibaculum*, and *Campylobacter* had a positive relationship with these serum lipids (Mu et al., 2020).

Yu et al. assessed the effect of naringenin on GM changes in HFD‐induced NAFLD among SPF C57BL/6J mice. They found improvement in the steatosis of liver cells, but intestinal permeability did not change significantly. Furthermore, liver weight, body weight, subcutaneous fat weight of the groin, epididymal fat weight, and brown fat weight were reduced in these animals. Blood and liver TG and TC were decreased after naringenin intake, and also intestinal and liver inflammation were attenuated by reduced gene expression of IL‐6, TNF‐α, IL‐1β, and F4/80. Myeloperoxidase activity (a clinical neutrophil enzyme that can produce aggressive oxidants) was decreased, too. GM was altered after naringenin intake. In particular, *Proteobacteria* in the phylum level; *Epsilonproteobacteria* in the class level; *Rikenellaceae*, *Helicobacteraceae*, and *Peptostreptococcaceae* in the family level; *Romboutsia*, *Alistipes*, *Faecalibaculum*, *Lachnospiraceae_NK4A136_group*, and *Desulfovibrio* in the genus level were positively related to above parameters. However, *Parasutterella* and *Bacteroidales*_S24‐7_group had a negative association with these parameters (Yu et al., 2023).

The studies showed naringenin supplementation modulates GM composition and gut barrier function. Inflammatory factors, lipid profile, liver enzymes, lipogenesis, and liver lipid accumulation improved. Therefore, naringenin may improve NAFLD, but more studies are necessary to confirm these findings.

### 4.5 Quercetin

Quercetin is a plant flavonol that is found in various vegetables, fruits, and grains, like onions, broccoli, and grapes (David et al., 2016). Despite its anti‐inflammatory and antioxidant activities, quercetin has anticancer, antidiabetic, and cardioprotective effects (Aghababaei & Hadidi, 2023; Zahedi et al., 2013). Also, it has antimicrobial activity, which can change the diversity of GM (Aghababaei & Hadidi, 2023; Lan et al., 2021). Quercetin effects as a GM modulator should be assessed on the gut‐liver axis (Table 1).

Shi et al. revealed the positive effects of dietary quercetin and isoquercetin on high‐fat‐induced NAFLD. At the microbial level, quercetin and isoquercetin had ameliorated effects on the composition of the GM. Analyzing showed that some bacteria from *Lactobacillus*, *Bifidobacterium*, and *Akkermansia* genera had a positive association with indole, indole‐3‐propionic acid (IPA), and indole acetic acid (IAA) in the feces which contribute to facilitating mucosal homeostasis and barrier function. Moreover, *Streptococcus*, *Anaerostipes*, and *Lachnoclostridium* had a negative association with fecal levels of IAA and IPA in mice (Shi et al., 2022).

Porras et al. assessed the effect of aglycone quercetin on NAFLD by changing the GM composition of HFD‐fed C57BL/6J mice. Consumption of aglycone quercetin decreased activation of (TLR‐4)‐NF‐κB pathway, expression of pro‐inflammatory genes, and reticulum stress pathway. Chronic endoplasmic reticulum stress elicits numerous intracellular pathways that have the potential to culminate in hepatic steatosis, systemic inflammation, and hepatocyte apoptosis. Aglycone quercetin intake improved gut barrier dysfunction, too. Regarding GM, it reduced the abundance of *Firmicutes/Bacteroidetes* and *Proteobacteria* ratio at the phylum level. At the class level, aglycone quercetin intake attenuated the abundance of *Deltaproteobacteria* and elevated the abundance of *Betaproteobacteria*, *Erysipelotrichi*, and *Bacteroidia*. Also, at the genus level, it elevated the abundance of *Sutturella*, *Allobaculum*, and *Flavobacterium* while reducing the abundance of *Helicobacter* and *Desulfovibrio* (Porras et al., 2017).

In the reported study by Juárez‐Fernández et al., male Wistar rats were used to evaluate the effect of quercetin alone or in combination with *Akkermansia muciniphila* on NAFLD. They found a significant decrease in plasma concentration of DCA and HDCA. Furthermore, the total primary BAs, the αMCA plasma concentrations, and the CA/DCA ratio were increased after the intervention. Hepatic expression of PPAR‐α, IL‐6, and IL‐1β were attenuated too. With regard to microbiota composition, they revealed that quercetin supplementation combined with *Akkermansia muciniphila* decreases the abundance of *Actinobacteria* phylum, and *Roseburia* and *Coprobacillus* genera, whereas it increases the abundance of *Cyanobacteria* phylum. Also, quercetin supplementation alone reduced the abundance of *Coprobacillus*. There was a significant positive relationship between *Actinobacteria* phylum and hepatic expression of IL‐1β. *Coprobacillus* genus was positively associated with hepatic expression of PPAR‐α, IL‐6, and plasma concentrations of DCA. However, the *Cyanobacteria* phylum was related to a higher CA/DCA ratio. *Roseburia* genus had a positive relationship with secondary HDCA and IL‐6, and a negative association with primary αMCA and primary BAs (Juárez‐Fernández et al., 2021). Based on studies, we discovered that quercetin intake affects the GM and intestinal barrier function, resulting in a positive impact on NAFLD. It has the potential to improve inflammatory factors and bile acids.

### 4.6 Resveratrol

Resveratrol (RSV) is a phytoestrogen, widely found in nuts, berries, and grapes naturally (Celotti et al., 1996; Lorenz et al., 2003). The advantageous effects of resveratrol include having anti‐inflammatory and anticancer properties, reducing cardiovascular diseases, preventing the oxidation of LDL‐Cs and platelet aggregation, and modulating lipid metabolism (Culpitt et al., 2003; Zhou et al., 2022). The potential influence of resveratrol metabolism by GM and its effect on microbiota composition showed a significant role caused by the interaction between RSV and the host microbiota in treatment efficacy (Bode et al., 2013; Theilmann et al., 2017).

Analyzing 19 mice with NAFLD, Du et al. conducted a significant NAFLD improvement after a 4‐week resveratrol supplementation via regulating GM. It reduced the abundance of *Firmicutes*, *Ruminococcaceae*, *Lachnospiraceae*, *Hydrogenoanaerobacterium*, *Anaerotruncus*, *Oscillibacter*, *Peptococcus*, *Clostridium XlVb*, *Flavonifractor*, *Intestinimonas*, and *Pseudoflavonifractor*. These bacteria were positively associated with HOMA‐IR, fasting blood glucose, liver TG, fat weight, body weight, and oil red O staining. However, *Bacteroidetes*, *Porphyromonadaceae*, *Allobaculum*, *Parasutterella*, and *Barnesiella* were increased after resveratrol intake, and were negatively correlated with the previous indicators. Furthermore, *Ruminococcaceae*, *Clostridium XlVb*, *Intestinimonas*, *Oscillibacter*, *Flavonifractor*, *Anaerotruncus*, and *Hydrogenoanaerobacterium* were negatively associated with phospho‐insulin receptor substrate 1 (p‐ IRS1) and also were positively associated with phospho‐mTOR (p‐mTor), mammalian target of rapamycin (mTOR), and PPAR‐γ (Du et al., 2021). The mTOR, p‐mTor, and PPAR‐γ contribute to lipid homeostasis (Feng et al., 2022; Parent et al., 2007; Walczak & Tontonoz, 2002).

Zhao et al. assessed the effect of resveratrol and polydatin (a glycoside of resveratrol) on high‐fructose diet NAFLD‐induced mice. They found that polydatin and resveratrol modify GM and increase fecal levels of valeric acid and caproic acid. There was a significant connection between higher valeric levels and the abundance of *Bifidobacterium* and *Butyricimonas*. Polydatin elevated the abundance of *Butyricimonas*, and both polydatin and resveratrol enhanced the abundance of *Bifidobacterium*. Also, both polydatin and resveratrol elevated the abundance of *Muribaculum* and *Desulfovibrio*. Furthermore, resveratrol enhanced the abundance of *Rinkenella* and decreased *Lactobacillus* (Zhao et al., 2022).

Chen et al. used NAFLD rats in their study to find the effect of resveratrol supplementation on NAFLD by changing GM composition. Resveratrol intake elevated the abundance of *Lachnospiraceae*, *Ruminococcaceae*, and *Akkermansia muciniphila* whereas reduced the abundance of *Desulfovibrio*. There was a significant positive relationship between mRNA expressions of cannabinoid receptor 2 (CB2), Occluding, and ZO‐1 in the distal colon and *Lachnospiraceae*, *Ruminococcaceae*, and *Akkermansia muciniphila*. However, *Desulfovibrio* was positively related to mRNA expression of cannabinoid receptor 1 (CB1) in colon and plasma LPS (M. Chen et al., 2020). CB2 receptor protects fatty liver by antifibrogenic and anti‐inflammatory signals, while CB1 increases fatty acid synthesis in HFD‐induced NAFLD (Mallat et al., 2011; Osei‐Hyiaman et al., 2005).

In another study, Wang et al. used C57BL/6 J mice to determine the effect of resveratrol on NAFLD. 300 mg/kg/day resveratrol supplementation with HFD after 16 weeks reduced liver oxidative stress, inflammation, body weight, and fat accumulation compared to the HFD group. Also, gut barrier permeability was improved too. Regarding GM, resveratrol intake enhanced the abundance of *Bacteroides*, *Allobaculum*, and *Blautia*, whereas it reduced the abundance of *Alistipes*, *Lachnospiraceae_NK4A316_group*, and *Desulfovibrio* (Wang et al., 2020).

Ke et al. assessed the effect of resveratrol and hydroxypropyl‐beta‐cyclodextrin (HBC) embedded resveratrol on SPF C57BL/6 mice. They found that resveratrol and HBC‐embedded resveratrol supplementation improves NAFLD via activating the AMPK pathway. In relation to GM, there was a significant positive relationship between *Bacteroidetes* and *Prevotella* and a negative association with *Firmicutes*, *Verrucomicrobia*, and *Facklamia*. Resveratrol and HBC‐embedded resveratrol intake reduced the abundance of *Verrucomicrobia* and *Firmicutes/Bacteroidetes* ratio, whereas elevated the abundance of *Bacteroidetes*. However, only HBC‐embedded resveratrol intake elevated the abundance of *Prevotella* and attenuated the abundance of *Facklamia* (Ke et al., 2023).

Using male C57BL/6J mice, Gao et al. analyzed the effect of resveratrol on NAFLD by regulating GM. Resveratrol declined fecal levels of *Alistipes*, *Desulfovibrio*, and *Lachnospiraceae_NK4A136_group*. *Desulfovibrio* had a positive relationship with liver TG, IL‐6, and mRNA levels of NF‐κB and TLR4. *Alistipes* was related to higher mRNA levels of TLR‐4 in the liver. Furthermore, the *Lachnospiraceae_NK4A136_group* positively associated with LPS, serum lipids, liver TG, liver IL‐6, and mRNA levels of NF‐κB and TLR4 (Gao et al., 2023).

Conversely, Milton‐Laskibar et al. found a different effect of resveratrol intake on the GM composition of male Wistar rats. NAFLD was induced by a high‐fat, high‐fructose diet in these animals. Also, they consumed 30 mg/kg/day of resveratrol for 8 weeks. The microbiome of rats was characterized by a greater presence of *Blautia* and *Lactococcus*, while these bacteria were positively correlated with increased liver weight, ballooning, steatosis, lobular inflammation, higher NAFLD activity score (NAS), and higher transaminase level (Milton‐Laskibar et al., 2021). The NAS incorporates potentially reversible traits of steatohepatitis (Juluri et al., 2011). The low dose of resveratrol (20 mg) may lead to the insignificant results of this study.

According to the studies, we found intake of resveratrol can modulate GM and intestinal barrier function with a positive effect on NAFLD. Oxidative stress, inflammatory factors, bile acids, glucose level, IR, and lipid profile were improved.

### 4.7 Sulforaphane

Sulforaphane is an aliphatic isothiocyanate known as an antibiotic. Glucoraphanin is the sulforaphane precursor in Brassica cruciferous vegetables like broccoli, cabbage, sprouts, and brussels (Vanduchova et al., 2019). When this vegetable is boiled, chopped, and chewed, glucoraphanin is altered into sulforaphane by a plant enzyme myrosinase (EC 3.2.1.147) (Shapiro et al., 2001). In the human body this process is also occurred after intake of the vegetables because the GM includes bacteria that produce myrosinase (Kaiser et al., 2021). Antidiabetic, anticancer, anti‐inflammatory, antioxidative, cardiovascular protective activities, and neuroprotective were reported as beneficial effects of sulforaphane (Greaney et al., 2016). Therefore, this substance may have advantageous effects in the treatment of NAFLD (Table 1).

Analyzing NAFLD mice, Xu et al. assessed the association of sulforaphane supplementation on NAFLD. A significant connection was seen between the intake of sulforaphane and reduced levels of liver and serum LPS, as well as downregulation of colonic endoplasmic reticulum stress genes and colonic inflammatory genes. Furthermore, the IL‐10/IL‐6 ratio in the liver and gene expression of ZO‐1 and Claudin‐4 were increased after sulforaphane consumption. Concerning GM composition, the relative abundance of *Butyricicoccaceae*, *Lachnospiraceae_NK4A136_group*, *Butyricicoccus*, *Allprevotella*, *Akkermansia*, and *Bifidobacterium* was correlated with a higher IL‐10/IL‐6 ratio in the liver and gene expression of Claudin‐4 and ZO‐1 proteins. Sulforaphane supplementation elevated the abundance of these bacteria. In contrast, sulforaphane attenuated the abundance of *Desulfovibrionaceae*, *Streptococcaceae*, *Lactobacillus*, *Desulfovibrio*, *Blautia*, *Lactococcus*, and *Firmicutes/Bacteroidota* ratio. They had a negative association with the IL‐10/IL‐6 ratio in the liver and the expression of ZO‐1 and Claudin‐4 genes, while they were positively associated with serum and liver LPS concentrations, colonic endoplasmic reticulum stress genes, and colonic inflammatory genes (Xu et al., 2023).

Xu et al. analyzed the effect of sulforaphane intake on NAFLD mice. 30 mice were separated into two groups, wherein one group was administered a HFD along with saline, while the other group received HFD supplemented with sulforaphane. IAA increased by changing GM composition. *Bacteroides* and *Bifidobacterium* are bacteria that produce IAA and were increased by sulforaphane. Also, sulforaphane increases *Firmicutes/Bacteroidetes* ratio and reduced *Deferribacteres* (Xu et al., 2021).

These studies showed that intake of sulforaphane can modulate GM and intestinal barrier function with a positive effect on NAFLD by reducing inflammation, but more studies are necessary to confirm these findings.

## 5 CONCLUSION

The findings from previous studies suggest that dietary supplementation with chlorogenic acid (CGA), curcumin, green tea catechins, naringenin, quercetin, resveratrol, and sulforaphane may have potential benefits in modulating the GM and intestinal barrier function, leading to positive effects on NAFLD. These supplements have shown promise in improving various factors associated with NAFLD, such as inflammatory markers, lipid profiles, liver enzymes, bile acids, insulin resistance, and liver fat deposition. While the results are encouraging, further comprehensive studies are required to validate and build upon these findings, as well as to elucidate the mechanisms underlying the observed effects. Additionally, the long‐term safety and efficacy of these dietary supplements in the management of NAFLD warrant further investigation. Furthermore, future research can focus on the potential synergistic effects of combining polyphenols with other dietary or therapeutic interventions for NAFLD and also clinical trials should be conducted to evaluate the effectiveness of polyphenol interventions in NAFLD patients.

## AUTHOR CONTRIBUTIONS

**Kimia Mohammadhasani:** Conceptualization (equal); data curation (equal); investigation (equal); writing – original draft (equal). **Mohammad Vahedi Fard:** Data curation (equal); investigation (equal); methodology (equal); writing – original draft (equal). **Ali Mottaghi Moghaddam Shahri:** Visualization (equal); writing – review and editing (equal). **Zahra Khorasanchi:** Project administration (equal); writing – review and editing (equal).

## FUNDING INFORMATION

No funds, grants, or other support was received.

## CONFLICT OF INTEREST STATEMENT

The authors declare that they do not have any conflict of interest.

## Data Availability

Data sharing not applicable to this article as no datasets were generated or analyzed during the current study.
